# COVID-19 and Pediatric Asthma: Clinical and Management Challenges

**DOI:** 10.3390/ijerph18031093

**Published:** 2021-01-26

**Authors:** José Laerte Boechat, Gustavo Falbo Wandalsen, Fabio Chigres Kuschnir, Luís Delgado

**Affiliations:** 1 Clinical Immunology Service, Internal Medicine Department, Faculty of Medicine, Universidade Federal Fluminense, Niterói 24070-035, Brazil; 2 Basic and Clinical Immunology Unit, Department of Pathology, Faculty of Medicine, University of Porto, Porto 4200-319, Portugal; ldelgado@med.up.pt; 3 Center for Health Technology and Services Research (CINTESIS), Faculty of Medicine, University of Porto, Porto 4200-319, Portugal; 4 Department of Pediatrics, Faculty of Medicine, Universidade Federal de Sao Paulo, São Paulo 04025-002, Brazil; gfwandalsen@uol.com.br; 5 Department of Pediatrics, Faculty of Medicine, Universidade do Estado do Rio de Janeiro, Rio de Janeiro 20943-000, Brazil; fabkuschnir@gmail.com

**Keywords:** COVID-19, SARS-CoV-2, childhood, pediatric, asthma, atopy, obesity, treatment, vaccines

## Abstract

Asthma is the most frequent chronic condition in childhood and a current concern exists about asthma in the pediatric population and its risk for severe SARS-CoV-2 infection. Although all ages can be affected, SARS-CoV-2 infection has lower clinical impact on children and adolescents than on adults. Fever, cough and shortness of breath are the most common symptoms and signs in children; wheezing has not been frequently reported. Published studies suggest that children with asthma do not appear to be disproportionately more affected by COVID-19. This hypothesis raises two issues: is asthma (and/or atopy) an independent protective factor for COVID-19? If yes, why? Explanations for this could include the lower IFN-α production, protective role of eosinophils in the airway, and antiviral and immunomodulatory proprieties of inhaled steroids. Additionally, recent evidence supports that allergic sensitization is inversely related to ACE2 expression. Obesity is a known risk factor for COVID-19 in adults. However, in the childhood asthma–obesity phenotype, the classic atopic Th2 pattern seems to predominate, which could hypothetically be a protective factor for severe SARS-CoV-2 infection in children with both conditions. Finally, the return to school activities raises concerns, as asymptomatic children could act as vectors for the spread of the disease. Although this is still a controversial topic, the identification and management of asymptomatic children is an important approach during the SARS-CoV-2 epidemic. Focus on asthma control, risk stratification, and medication adherence will be essential to allow children with asthma to return safely to school.

## 1. Introduction

In December 2019 a new infectious disease started in Wuhan in the Hubei Province of China, as a cluster of severe pneumonia cases, at first of unknown etiology. Severe acute respiratory syndrome coronavirus 2 (SARS-CoV-2), previously known as 2019-nCoV, is the cause of Coronavirus Disease 2019 (COVID-19) [1,2]. The first case series in 10 children were reported by Jiehao et al. and date back to January 2020 [3]. On March 11, 2020, when SARS-COV-2 infection had reached more than 100,000 people, over 4000 lethal cases, in more than 100 countries around the world, the World Health Organization (WHO) classified the outbreak as a pandemic [4]. At the time of this writing, more than 90,335,000 confirmed cases and over 1,954,000 deaths associated with SARS-CoV-2 infection have been reported around the world [5].

All epidemiologic data until now suggest that SARS-CoV-2 infection is less severe and prevalent in children than in adults. An alternative explanation for this fact is that COVID-19 has not been diagnosed that often in children because many in this age group remain asymptomatic [1]. The main questions about COVID-19 in the pediatric age group (<19 years) are: why the disease is predominantly mild in children? In addition to, as COVID-19 predominantly affects the lungs, can we consider chronic lung diseases, such as asthma, a risk factor in children where this respiratory disease is highly prevalent? 

The main objective of this narrative review is to address the clinical, epidemiological and immune response of COVID-19 in children—with a focus on asthma—in attempt to answer, with the available evidence to date, questions regarding the lower severity of SARS-CoV-2 infection in this age group and the interrelationships with other factors, such as atopy and obesity. Additional aspects around the therapeutic approach to children with asthma in times of COVID-19, herd protection, vaccination and returning to school are also discussed. 

## 2. Methodology

To retrieve information for this review, the authors searched PubMed, preprint servers (MedRxiv and BioRxiv), and selected publications cross-references, from January 2020 to January 2021, using keywords including “COVID-19”; “SARS-CoV-2”; “childhood”; “pediatric”; “asthma”; “atopy” and “obesity”. The inclusion criteria were: any type publication on COVID-19 related to children and asthma, written in English, Spanish or Portuguese. For this narrative review, evidence was included from systematic reviews and meta-analysis studies, randomized clinical trials, original research and observational studies, case series, position statements and selected reviews addressing the covered questions or cross-references from these publications.

There are several limitations to the available evidence for this narrative review: first, the information regarding asthma and COVID-19 in childhood is still limited; second, there are still insufficient data on the risk and protective factors in children [6,7,8]; finally, our understanding of the disease is rapidly evolving, and what the current evidence supports today, may soon change with the accumulation of new knowledge of SARS-CoV-2 biology and the host immune response.

## 3. The Clinical Presentation and Epidemiology of SARS-CoV-2 Infection in Children

### 3.1. Main Clinical Presentations

Consistent with observations during the past outbreaks of SARS-CoV-1 (2002) and MERS (2012), children with SARS-CoV-2 infection are more likely to have an asymptomatic or mild illness, with a significantly lower mortality than is seen with adult infection globally [9]. Asymptomatic infections have been reported in 4% of virologically confirmed cases [10], and in 20–30% of pediatric patients in different clinical series [11,12]. Most likely, these numbers are actually expected to be higher, as asymptomatic children are not systematically tested, and ongoing and future population-based seroprevalence surveys will be required to understand the true prevalence of asymptomatic infections.

Similar to adult patients, the proportion of female and male patients is comparable in the pediatric/neonatal population [13]. Although there are variations between different patients’ series, the main presentation symptoms reported in children have been similar to those in adults: fever (40–80%), cough (50–80%), shortness of breath (13–30%), rhinorrhea/nasal congestion (4–35%), sore throat or pharyngeal erythema (5–50%), headache (3–28%) and myalgia (10–25%) [9,14,15]. Among symptomatic children, only 5% presented with dyspnea or hypoxemia, and 0.6% progressed to acute respiratory distress syndrome (ARDS) or multi-organ system dysfunction [10]. Moreover, preschool children and infants are more likely to have severe clinical manifestations [16,17]. 

Of note, even when present, the symptoms of pediatric SARS-CoV-2 infection are rather non-specific, and overlap considerably with non-COVID-related illnesses, such as other viral respiratory tract infections commonly seen in children, such as influenza and respiratory syncytial virus (RSV). In practice, relying on the presence of fever or other respiratory symptoms may be ineffective for the diagnosis of COVID-19 in childhood.

Loss of smell is a well-recognized diagnostic symptom of COVID-19, that has been reported by otherwise asymptomatic patients, making it useful for the initial clinical diagnosis [18]. Although data on anosmia and ageusia in children and adolescents with SARS-CoV-2 infection are scarce and often under-reported, especially in children under 3 years old [17], these symptoms can also occur alone or in rather asymptomatic patients in this age group [19]. Other reported clinical presentations in children are gastrointestinal symptoms (abdominal pain, nausea/vomiting and diarrhea) and it is interesting to note that the onset with gastrointestinal symptoms is a marker of a more severe clinical condition [20]. Cutaneous findings include chilblain-like lesions (“COVID toes”), vesicular eruptions, urticarial lesions, maculopapular eruptions and livedo or necrosis, although the causal link between these dermatologic presentations and SARS-CoV-2 infection are still unclear [14]. Several reports have shown that children tend to have more upper respiratory tract involvement, rather than involvement of the lower respiratory tract. Wheezing reports have not been frequent in children with COVID-19, as well as ocular manifestations of conjunctivitis [14]. The prevalence of neurological disorders and coagulopathy with thrombosis in children with COVID-19 is not yet known to date (Table 1).

Recently, the concept of “long COVID-19” has been discussed in the literature. This terminology, not yet clearly defined, suggests some of the characteristics of chronic fatigue syndrome, referring to the maintenance of continuous and debilitating symptoms, such as chest heaviness, breathlessness, muscle pains, palpitations and fatigue, often months after the onset of the disease. Currently, it is not yet clear who tends to suffer from this condition, but it has been described even in previously healthy young people [21,22]. 

### 3.2. Multisystem Inflammatory Syndrome Presentation in Children

Although acute SARS-CoV-2 infection tends to be mild or symptom-free in most pediatric cases, several reports of a multisystem inflammatory syndrome in children (MIS-C), temporally associated with SARS-CoV-2, with overlapping features of atypical Kawasaki disease (KD), toxic shock syndrome (TSS) and macrophage activation syndrome (MAS), have started to appear in the literature [23,24]. The CDC case definition for MIS-C comprises: age < 21 years, fever, severe illness with two or more organ systems affected, laboratory evidence of inflammation, laboratory or epidemiologic evidence of SARS-CoV-2 infection and no other alternative diagnosis [25]. Cytopenias (lymphopenia, thrombocytopenia) distinguished MIS-C from KD and the degree of hyperferritinemia (significantly higher in MAS compared with MIS-C) and the pattern of cytokine production (IL-18, CXCL9 and soluble IL-2 receptor levels are greatly increased in MAS) differs MIS-C from MAS [26].

Reports from Italy [27], the UK [28], the US [29] and France [30] noted a delay of 30–45 days between signs of COVID-19-like illness (or contact with someone with known or presumed COVID-19), and the onset of inflammatory syndrome, as a common pattern in these patients. The pathogenesis of MIS-C is unknown, but several observations point to a possible post-infectious, delayed immunologically mediated phenomenon or autoimmune disease, following either symptomatic or asymptomatic SARS-CoV-2 infection [31], raising concerns of possible immune-mediated adverse effects related to the future vaccination in children (see below). Several hypotheses have been proposed for the MIS-C pathogenesis: an inefficient and reduced neutralizing antibody activity against SARS-CoV-2, leading to immune enhancement following SARS-CoV-2 re-exposure [32]; a direct effect of SARS-CoV-2 Spike protein acting as a superantigen (in a similar way to *Staphylococcal Enteroxin B*) and mediating hyperinflammation [24]; and an autoimmune response related to molecular mimicry, where SARS-CoV-2 antigens share sequence or structural similarities with self-antigens, both at the cellular and humoral level, following infection (and vaccination?) and leading to production of autoreactive antibodies [33].

### 3.3. Radiographic Characteristics

The chest radiographic changes reported in children are also generally less pronounced than in adults [34]. A systematic review that analyzed chest computed tomography (CT) in subjects under 18 years old, showed that 34% of the imaged cases were normal [35]. The most characteristic pattern described was “ground glass” opacifications in 62.3% of the CTs. The changes were mostly located in the lower lobes (44.4%), unilateral in 53.4% of children and classified as mild [35]. Underlying coinfection may be more common in children than in adults, and consolidation with surrounding halo sign is considered a typical sign described in 9.4% of pediatric patients [35,36]. Pleural effusion was rare (1.2–2.0%) [35,37]. Imaging appearances at follow-up (3 to 15 days after admission) remain normal or improve in the majority of children [35]. Of note, some children remain asymptomatic, even when they have radiologically detected pneumonia [10].

### 3.4. Laboratory Examination Results

The lymphocyte count was below the normal level in only 15% (95% CI: 8–22%) of children, which is lower than in adult patients [38,39]. Normal white blood cell counts were observed in 69% (95% CI: 64–75%) and elevated D-dimer, C-reactive protein, lactate dehydrogenase (LDH), procalcitonin and creatine-kinase MB in, respectively, 11% (95% CI: 8–14%), 19% (95% CI: 13–26%), 29% (95% CI: 20–39%), 36% (95% CI: 21–51%) and 37% (95% CI: 25–48%) of pediatric patients [37].

The subset of children who develop severe COVID-19 disease requiring pediatric intensive care unit (ICU) admission have been reported to have significantly higher markers of inflammation (C-reactive protein, pro-brain natriuretic peptide, procalcitonin) compared with patients admitted to general pediatric medical units [40]. Children infected with severe COVID-19 have also shown a significant decrease in T-cell subsets and low levels of C3 and C4 in the acute stage of the disease [20].

In MIS-C patients, laboratory measurements have shown an elevated mean neutrophil percentage (80.7 ± 7.8%), while the mean lymphocyte percentage was low (9.8 ± 0.8%). C-reactive protein (160 ± 6.9 mg/L), ferritin (977 ± 55.8 ng/mL), and procalcitonin (30.5 ± 2.1 ng/mL) were markedly increased. Cardiac markers, troponin, brain natriuretic peptide, and prohormone of brain natriuretic peptide, were extremely elevated at 494 ± 37.6 ng/L, 3604 ± 352 pg/mL, and 5854 ± 743 ng/L, respectively [41].

## 4. Why Is COVID-19 So Mild in Children?

The reasons for the lower severity and incidence of the disease in children is still unclear [42]. Some hypotheses have been raised to explain these findings, based on possible different risk and protective factors compared to adult patients.

### 4.1. Less Efficient Internalization Process of the Virus

Different studies have shown that the expression of angiotensin-converting enzyme 2 (ACE2), the main receptor for the SARS-CoV-2 spike protein, in the upper airways is age-dependent and expressed at a lower level in children than in adults [43,44]. Interestingly, Yonker et al. [9] demonstrated that higher ACE2 expression correlates with being positive for SARS-CoV-2 genomic RNA in swabs of upper respiratory tracts from symptomatic children, but not with the viral load. In addition to the presence of ACE2, the entry of SARS-CoV-2 into cells is dependent on the transmembrane serine protease 2 (TMPRSS2), which cleaves the spike protein into two subunits, allowing viral fusion with cell membrane [45]. Both receptors are widely distributed in epithelial barrier sites, such as the nose, lung, heart, kidney and intestine, but rarely expressed in cells of the immune system [46]. However, it has recently been shown that epithelial cells, macrophages, monocytes, innate lymphocytes (ILCs), NK cells, T cells and B cells can be infected or carry SARS-CoV-2 through their binding to CD147—a receptor not exclusive to SARS-CoV, but also present in HIV-1 and measles—and contribute to the local and systemic spread of the virus [47]. Radzikowska et al. [48], showed that as well as ACE2, several CD147-related genes, such as CD44, CD98, monocarboxylate transporters (MCTs), glucose transporter GLUT1 (SLC2A1) and NFATC1 (nuclear factor of activated T cells) showed lower expression in peripheral blood mononuclear cells and T cells in children. Collectively, these findings suggest that in the younger age groups the internalization process of the virus is less efficient, and may result in milder clinical manifestations or even a greater number of asymptomatic patients. Additionally, the known risk factors for severity and mortality of COVID-19 in adults, such as cardiovascular disease, diabetes, cancer, immunosuppression, smoking, are not prevalent or are absent in children [6].

### 4.2. Viral Co-Infection

As previously described, children with COVID-19 present distinct clinical characteristics from adult patients. In addition to a lower severity, the rate of co-infection with other respiratory viruses in paediatric patients is notably higher than in adults [49]. Experimental models of respiratory virus co-infections have demonstrated diverse interactive effects, which can lead to cooperative or competitive interactions, with a consequent increase or reduction in viral growth [50]. These interactions have also been postulated in epidemiological studies. During the influenza A virus pandemic in 2009, interactions between this microorganism and other respiratory viruses, such as rhinovirus and RSV, may have delayed the dissemination of the pandemic virus in Europe [51], while in turn it may have interfered with RSV epidemics in the same period [52]. In this context, it would be plausible to speculate that the simultaneous presence of different viruses in the airways of young children could restrict the development of SARS-CoV-2 by competition or interaction and mitigate the severity of the disease. However, it is still not clear if SARS-CoV-2 and other respiratory pathogens have a similar mode of interaction. Ma et al. [53] evaluated 250 adult patients diagnosed with COVID-19 who visited a hospital in Wuhan, China. No statistically significant difference was found in age, sex, the time needed to return negative SARS-CoV-2 nucleic acid test results, length of stay and mortality between the group of isolated SARS-CoV-2 infection and the group with co-infection. On the other hand, Götzinger et al. [34], in a European multicenter study, demonstrated that viral co-infection was associated with a higher risk of ICU hospitalization. Further studies involving children are needed to better understand the possible mechanisms of interaction and competition from other respiratory pathogens in SARS-CoV-2 infection.

### 4.3. Influence of Comorbidities on Pediatric Presentations

Risk factors for severe manifestations of COVID-19 include, among others, underlying comorbidities such as diabetes, cardiovascular disease and obesity [54]. Although early studies have established pre-existing comorbidities as significant risk factors for severe SARS-CoV-2 infection in adults, some recent studies addressed the relation of childhood comorbidities and COVID-19 outcomes [7,8]. A systematic review showed that among pediatric patients with SARS-CoV-2 infection and underlying comorbidities, 5.1% had severe COVID-19 and/or were admitted to the pediatric ICU, while this occurred in only 0.21% of pediatric patients without comorbidities (RR of 1.79 (95% CI 1.27–2.51)) [7]. The risk ratio of mortality among children with comorbidities in comparison with pediatric patients without comorbidities was 2.81 (95% CI 1.31–6.02). The main comorbidities observed in children with severe COVID-19 were obesity, chronic respiratory disease, cardiovascular disease, neurologic disorders, immune disorders and metabolic disease [7,8]. In conclusion, children with obesity and chronic respiratory disease have a predisposition to critical illness following infection with COVID-19, although the absolute risk remains low. The immune mechanisms underlying enhanced resistance to infection and a reduced risk of progressive disease (see below) appear to protect most children with comorbidities from severe disease [8].

### 4.4. A Protective Innate Immunity

As SARS-CoV-2 is a pathogen so far not recognized in humans, it would be expected that the triggering of an effective adaptive immune response to neutralize new antigens (i.e., a primary immune response of antibodies and/or T cells) would operate around the second to third week of viral contact. This timing seems to indicate that infection control, in asymptomatic or mild patients, will probably be due to the innate or nonspecific first line immune response, whose activation does not depend on recognition by antibodies and/or T lymphocytes [2,55].

The innate immune system provides an early first line of defense, essential in defending against invading new pathogens, mainly cytopathic viruses. The interferon (IFN) response is a first line viral defense and—for many respiratory viruses, including SARS-COV-1 and MERS-CoV, IFN types I and III (so-called “innate” interferons)—appears to play a relevant role in limiting infection [56].

Antiviral innate immunity encompasses several other humoral components, such as those of the complement and coagulation-fibrinolysis systems, soluble proteins that recognize glycans on the cells surface (e.g., Mannose Binding Lectin (MBL)), and the so-called natural antibodies (IgM, but also IgA and IgG). It also includes cellular components, including NK cells, ILCs and gamma delta T cells, which generally limit the spread of viral infection by their cytotoxic action on target cells, the production of cytokines and, ultimately, promotion of an adaptive response (from T and B lymphocytes) [57]. Therefore, an imbalance of the innate immune response is possibly one of the initial factors promoting viral proliferation and immune dysregulation. As aging is a risk factor in this way (natural antibodies and serum MBL levels decrease significantly with age), a protective role of innate immunity may be one of the explanations for mild disease in childhood, as outlined above [58,59].

Still in this context, children may develop a more efficient production of neutralizing natural antibodies against SARS-CoV-2 at the beginning of the infection due to the greater number of activated B cells of IgM + memory [60]. Moreover, they may have a larger thymic repertoire and more T- and B-regulatory lymphocytes, in addition to a more effectively trained and sustained innate immunity, due to increased natural exposure to viruses and vaccines [61,62]. In this way, frequent infections and vaccines/adjuvants administered in early life may booster innate immunity in childhood [58,59], resulting in a more protective immune response in children.

### 4.5. Is There a Role for T Cell Mediated Immunity?

Grifoni et al. [63] measured SARS-CoV-2-specific CD4+ and CD8+ T cells responses in 20 COVID-19 cases and healthy control donors. CD4+ T cell and antibody responses were observed in all COVID-19 patients analyzed, and CD8+ T cell responses in most. In this same study, CD4+ T cell responses were also detected in 40–60% of unexposed individuals. It has been suggested that SARS-CoV-2-specific T cells in unexposed individuals might originate from memory T cells to cross-reactive epitopes derived from exposure to common cold human coronaviruses, which widely circulate in the human population with mild self-limiting respiratory symptoms. Whether this previous immunity is relevant in influencing clinical outcomes in childhood is unknown, but it may be of value in herd immunity and vaccine development (see below) [64].

Another issue under intense debate is the role of T cells in the protection against SARS-CoV-2 reinfection [65,66]. Some authors state that SARS-CoV-2 reinfection seems unlikely, taking into account knowledge about viral neutralization and antibody duration in previous respiratory diseases. Thus, false-positive cases of reinfection may have occurred mainly due to technical errors associated with the different steps in the collection of samples and in the analysis of the RT-qPCR swab test or by prolonged viral spread, rather than reinfection [67,68]. However, due to the lack of sufficient data, one must take into account the probability of genetic mutations favoring new infection, instead of reinfection by the same lineage, before considering these patients as not infected [67]. Additional multicenter cohort studies, including children, are required for a better understanding of the potential for the recurrence of SARS-CoV-2 infection, and how the immune system works in this situation [68].

### 4.6. Is There an Influence of a T2 Response?

Kimura et al. [69] demonstrated that type 2 inflammation (IL-4 and IL-13) reduces ACE2 and increases TMPRSS2 expression in nasal and bronchial epithelial cells in asthma and atopy. These ex vivo observations are supported by the analysis of two databases, which included children and adults with type 2 rhinitis and asthma, and showed similar results. In contrast, ACE2 receptors are upregulated by type 1 IFNs, suggesting that Th1/Th2 balance may influence the course of SARS-CoV-2 infection [70]. Children have less potent pathogen-associated molecular patterns (PAMP) activation, suboptimal and Th2 skewed cytokine production, all possibly resulting in a lower inflammatory immune response. This confers decreased protection against infection during childhood, but seems beneficial in the prevention of an inflammatory response in COVID-19. Hence, a preferential Th2-skewed cytokine production observed in children is presumably protective against SARS-CoV-2 infection [71].

### 4.7. Herd Immunity—Is It Favoured by Asymptomatic Infection in Children?

Conceptually, the term herd immunity (or herd protection) refers to the population’s resistance to the spread of a contagious disease and was first proposed by Topley and Wilson in 1923 [72]. It only exists when a sufficient high proportion of the population generate immunity against the foreign pathogen, making it difficult for the contagious disease to spread between individuals, and protecting susceptible individuals from infection [73]. It can be achieved in two ways: having the disease (and recovering from it) or by wide-scale vaccination. In general, it is accepted that about two-thirds of a population must be immunized to achieve herd immunity [74], and this threshold seems to apply also to COVID-19 [73,75]. Much has been said about asymptomatic or oligosymptomatic SARS-CoV-2 infections in childhood favoring the natural establishment of herd immunity, but so far there is no evidence to point in this direction. What we know is that children probably play a relevant role in SARS-CoV-2 transmission and infection dynamics, and are possibly a critical subgroup for the effective control of outbreaks [14].

Another unanswered issue, is if the rapid establishment of a global herd immunity through mass vaccination using an appropriate vaccine could eliminate SARS-CoV-2. In many infectious diseases, vaccination of children to induce herd immunity has proven successful in preventing the disease spread and/or severity, for example against influenza and pneumococcal disease. Nevertheless, the relevance of children in SARS-CoV-2 transmission is still unclear at this point, in particular, whether asymptomatic children and those with low viral loads play a significant role in transmission networks [76].

## 5. Pediatric Asthma and SARS-CoV-2 Infection

Human coronaviruses (hCoVs) circulate globally, commonly infecting children and generally causing mild, self-limiting upper respiratory tract infections (rhinorrhea, nasal congestion, sore throat and fever). Asymptomatic infection also occurs. Of the seven coronaviruses identified in humans, hCoV-229E and hCoV-NL63 belong to α-coronaviruses, and hCoV-OC43, MERS-CoV, SARS-CoV-1 and SARS-CoV-2 belong to β-coronaviruses. Both SARS-CoV-1 and SARS-CoV-2 first emerged in China [14].

Up to 80% of asthma exacerbations are due to viral infections, including common coronaviruses [77]. Two large population based-studies in hospitalized children, one in Norway [78] and the other in China [79], found that, respectively, 10 and 4.3% of hospitalized children tested positive for hCoVs, with the most common type being hCoV-OC43 (a β-coronavirus) in both studies.

This fact draws attention to the possibility of these previous infections with hCoVs in children conferring partial protection against SARS-CoV-2. Despite lower respiratory tract infections with hCoVs have been described, severe illness is rare in the absence of additional risk factors (age < 12 months, immunodeficiency, presence of previous chronic pulmonary disease and co-infection with other pathogens) [79,80]. No clear association between hCoVs infections and Kawasaki disease has been identified [81,82].

Children infected during the SARS-CoV-1 epidemic were sporadic (<5% of confirmed cases) and had a clear history of exposure. The most common symptoms were fever (98%) and cough (60%), and the majority of children have a good prognosis. However, age > 12 years predicted a worse outcome [83]. The most common laboratory abnormalities in SARS-CoV-1 were similar to those seen in SARS-CoV-2, and included lymphopenia, thrombocytopenia and elevated LDH. Regardless of age, sore throat and elevated neutrophil count at presentation were independent risk factors for severe illness. Radiographic abnormalities consisted primarily of ground-glass opacities and/or areas of consolidation (similar to COVID-19) [84]. However, no cases of SARS-CoV-1 have been reported since early 2004.

MERS data on prevalence, clinical presentation and outcome in childhood are scarce. The most common symptoms in children were fever, cough, shortness of breath and vomiting/diarrhea. In chest radiographs, diffuse bilateral infiltrates were the most common abnormality [85].

The clinical and laboratorial data described above reinforce the idea that: (1) pre-existing cellular immunity to SARS-CoV-2 in unexposed patients (mainly children) may drive from previous exposure to beta hCoVs (OC43 being the most likely candidate) and (2) a great similarity (79%) exists between SARS-CoV-1 and SARS-CoV-2, as demonstrated by genetic analysis, which may reflect in the pathophysiology and clinical/laboratorial manifestations of both diseases [64,86].

### 5.1. Asthma and COVID-19

Two questions remain crucial regarding the association of asthma and COVID-19. Are asthmatic patients more susceptible to be infected by SARS-CoV-2? If infected, are asthmatic patients at higher-risk of severe COVID-19? Another question revolves around if the relationship between asthma and COVID-19 is different according to age (adults vs. children) and asthma endotypes (eosinophilic vs. non-eosinophilic asthma).

In a recent review, Skevaki et al. [87] found large country-differences in asthma prevalence among patients (mainly adults) with COVID-19. In most countries, such as China, Brazil, India, Mexico, Saudi Arabia, Spain and Italy, the prevalence of asthma among COVID-19 patients is lower than that observed in the general population, while the opposite is observed in the USA, Australia, UK and Ireland. The lack of more detailed information about asthma severity and phenotypes in most of these studies limits further conclusions.

Data in children are very scarce. Du H et al. [54] reported data from 182 children with COVID-19 hospitalized in Wuhan; although 23.6% had some form of allergic disease, only one child had asthma. In this study, no difference in clinical symptoms, severity and complications was observed between those with and without allergy [54]. On the other hand, in Brazil, asthma was found to be the most common comorbidity among 115 children with COVID-19 [17]; nevertheless, the prevalence of asthma among infected children was lower than that found in local epidemiological studies (13.0% vs. 20–25%) [88] and asthma was not associated with more severe COVID-19 leading to hospitalization. Data from 46 children admitted due to COVID-19 in a New York hospital showed that asthma was a frequent comorbidity (24%), but was not associated with the need for intensive care treatment [40].

Some studies have reported the effects of the COVID-19 pandemic among asthmatic children. In a recent online survey of 91 asthma experts, caring for more than 133,000 asthmatic children in five continents, only 14% of the responders reported suspected cases of COVID-19 among their patients; asthmatic children had mild symptoms in 73% of the cases and only one child required hospitalization [89]. Ruano et al. [90] described clinical data from 29 allergic asthmatic children with probable COVID-19 (suggestive symptoms in the child and in one adult living in the house) from a single center in Madrid, Spain. All children had mild symptoms of COVID-19 and there were no hospitalizations. Mild bronchospasm was observed in 24%, and oral corticosteroids were prescribed in only one case.

Severity and complications of SARS-CoV-2 infection are associated with hyper-inflammation. In asthmatics such hyper-inflammation could be down regulated by several mechanisms including the delayed and inefficient antiviral response due to lower IFN-α production by dendritic and epithelial cells, protective role of eosinophils in the airway, and antiviral and immunomodulatory proprieties of inhaled steroids [91]. Interesting, according to these explanations, such mechanisms that contribute to higher morbidity and higher lung involvement in most respiratory viral infections in asthmatics, are the same ones that are responsible for protecting them during SARS-CoV-2 infection.

In a systematic review of the literature, Castro-Rodriguez et al. [6] concluded that there are almost no data to assess whether asthma, or other pediatric respiratory diseases, constitutes a risk factor for SARS-CoV-2 infection or the severity of COVID-19, and emphasized the need for studies to address this issue in childhood. These authors discuss whether the lower prevalence of asthma among cases of COVID-19 may be due to a bias, since patients with chronic lung diseases may be more cautious when practicing social distancing and other measures to prevent infection, or even more reluctant to seek medical attention, even when sick, and so not being accounted for in health statistics [6].

In the real world, COVID-19 has not been implicated as an important driver of viral wheeze or asthma exacerbations in children. According to data from some hospitals, the number of presentations and/or admissions due to wheezing/asthma during the COVID-19 pandemic were either similar or lower than that observed in previous years [92,93]. Within this context, Abrams et al. [94] argue that although non-epidemic coronaviruses are commonly found in the respiratory tract of children, with exacerbation of asthma and contribution to bronchial hyperreactivity and eosinophilic inflammation, paradoxically, asthma exacerbations actually decreased during the COVID-19 epidemic, which can be attributed to greater attention being paid in relation to hygiene measures in this population. Obviously, the impact of the generalized lockdowns must be considered as a confounding factor.

Although studies on asthma and COVID-19 in children have so far been reassuring, the European Academy of Allergy and Clinical Immunology (EAACI) declared that “based on common sense, rather than mounting evidence” children with asthma, particularly severe or uncontrolled, should be considered to be at increased risk of developing severe COVID-19 [95].

### 5.2. Risk and Protective Factors Associated with Pediatric Asthma

#### 5.2.1. Atopy

Several mechanisms have been proposed to explain the lower morbidity of COVID-19 observed in patients with type 2 asthma.

As previously described, ACE2 is a host molecule used in cell entry by SARS-CoV-2 and other coronaviruses [69,96]. Data from three different cohorts of children and adults have shown that asthma and respiratory allergy were associated with lower expression of ACE2 gene in airway cells [96], as type 2 inflammatory mediators (such as IL-13) and allergen exposures may decrease ACE2 expression in bronchial epithelial cells [69,96].

The South Korean nationwide cohort tested almost 220,000 adults for SARS-CoV-2, with 7340 positive cases, and information regarding asthma, allergic rhinitis and atopic dermatitis was obtained from the health insurance records. Asthma was associated with an increased risk of SARS-CoV-2 test positivity (12.7% vs. 7.6%) and severe COVID-19 infection (ICU admission, mechanical ventilation, or death: 6.9% vs. 4.5%). Interesting and of note, was the report that both risks were higher in non-allergic asthma, with no significant risk being found when allergic asthma was compared to the non-infected group [97].

Du Y et al. [98] reported that about 80% of patients who died of COVID-19 had eosinopenia, a finding considered as a biomarker of poor prognosis of the disease and one of the best predictors for the severity when screening patients with COVID-19 [99]. It has been speculated that the eosinopenia was independent of the use of corticosteroids, and is related to the depletion of CD8+ T cells and the consumption of eosinophils caused by SARS-CoV-2 [61]. On the other hand, a minority of patients with COVID-19 have eosinophilic inflammation, suggestive that a type 2 inflammatory predominance plays a protective role against SARS-CoV-2 [100].

#### 5.2.2. Obesity

Obesity has been identified as an independent risk factor for serious disease and fatal outcomes in adults with COVID-19 [101]. Possible mechanisms for these findings include endothelial dysfunction associated with metabolic syndrome, increased expression of SARS-CoV-2 entry receptors ACE2, TMPRSS2 and CD147 in adipose tissue, and deficient pulmonary mechanics [102,103]. Interestingly, in a cohort of adults with COVID-19 it was reported that CD147 expression in the whole blood correlated positively with body mass index (BMI), and its upregulation by high glucose concentrations, which might reflect a correlation with obesity and also potentially with diabetes, another relevant COVID-19 comorbidity [48]. In contrast to other risk comorbidities for COVID-19 in adults, obesity is nowadays a worldwide epidemic in children [104]. Asthma and obesity are among the most prevalent diseases of children, and both are pro-inflammatory conditions. Systemic inflammation, related to excess visceral adiposity, has been identified as a possible mechanism for the development of the obesity–asthma endotype [105,106].

Although considered as factors of greater susceptibility and severity for COVID-19, studies in adults and children with both conditions have shown controversial results [107]. Most studies on asthma and COVID-19 were carried out in elderly people with many comorbidities as obesity, hypertension and diabetes, not clarifying whether asthmatic patients with COVID-19 have isolated asthma or asthma as a multimorbidity [108]. Thus, studies focused on asthma–obesity comorbidity are needed to establish the real role of asthma as a risk or protective factor in obese patients with COVID-19 in different age groups.

A study conducted by Lovinsky-Desir et al. [102] in New York City (NY), with the aim of determining whether asthma was associated with unfavorable outcomes, evaluated 1298 patients, 65 years or younger, hospitalized with severe COVID-19. The authors concluded that outcomes did not differ between obese and non-obese patients, with and without asthma, suggesting no change in risk attributed to asthma alone [102]. In another study, involving 1747 children and adolescents who visited an NY emergency department, 67 (34.5%) tested positive for SARS-CoV-2 infection, with 46 subsequent hospital admissions, and 13 (28.3%) patients needing ICU. Obesity and asthma were highly prevalent but not significantly associated with ICU admission [40].

The endotype associated with the characteristic late-onset adult obese asthma phenotype, might be an additional risk factor for COVID-19. Studies in obese asthmatic adults have predominantly shown a Th1/Th17 pattern in the airways´ inflammation, with a significant neutrophilia, associated to elevated levels of TNF-α, IFN-γ and IL-6 [109]. On the other hand, with the childhood asthma–obesity phenotype, the “classic” atopic Th2 pattern seems to predominate, with eosinophilic inflammatory infiltrate and high levels of IL-4, IL-5 and IL-13, which could hypothetically be a protective factor for severe SARS-CoV-2 infection in children with both conditions [110].

### 5.3. Management of Asthmatic Children during the SARS-CoV-2 Pandemic

Objective evidence regarding the optimal management of asthmatic children during the SARS-CoV-2 pandemic is still limited and most available information is based on expert recommendations. Weak evidence supports that some asthma medications such as inhaled steroids, montelukast and bronchodilators may have SARS-CoV-2 and/or other coronavirus inhibitory action, or could be beneficial to reduce COVID-19-driven inflammation [111,112,113].

An ex vivo study of an asthmatic’s airway epithelial cells showed that inhaled steroids have suppressive effects on ACE2 and TMPRSS2 expression [114]. A systematic review found no evidence of COVID-19-related adverse outcomes in patients with continuous or pre-morbid inhaled steroid use [115]. This evidence reinforces the recommendation from the Global Initiative for Asthma (GINA) and others that control medication for asthma, including inhaled steroids, should be maintained during the pandemic [116,117]. The decision to reduce or to step down daily controller medication should be carefully considered [117]. Oral corticosteroids were initially not recommended for patients with COVID-19 due to the potential risk of immune depression and a worse viral response [118,119]. However, further studies have shown beneficial effects of systemic corticosteroids in reducing acute respiratory distress syndrome and systemic inflammation [120]. For asthma exacerbations, a short course of oral steroids is recommended—according to clinical judgment—to prevent serious consequences [116,119].

Biologicals for type 2 severe asthma should also be maintained with self-application whenever possible [121]. Nevertheless, in cases of SARS-CoV-2 infection, suspension of biologicals is recommended until clinical recovery and viral clearance [121].

Management of comorbidities to optimize asthma control, mainly rhinitis, may be especially relevant during the COVID-19 pandemic. Uncontrolled rhinitis may mimic viral infection and possibly increase the risk of viral transmission in those infected with SARS-CoV-2 [122].

Routine spirometry testing should be suspended [116]. Telehealth and virtual appointments should be stimulated, and face-to-face visits should be limited to severe or uncontrolled patients [117].

Public health measures, as wearing facemasks and maintaining social distancing, if possible, are desirable. There is no evidence that wearing a facemask exacerbates asthma or any other underlying lung condition [122]. The CDC recommends for everyone, but particularly for patients with asthma, to avoid crowds, wash hands often with soap and water or use hand sanitizer that contains at least 60% alcohol, avoid cruise travel and non-essential air travel, stay home during a COVID-19 outbreak in the community and avoidance of people who are sick [123].

## 6. Return to School

The return to school activities of children and adolescents after the slowdown in lockdown has become a major challenge for epidemiologists worldwide [42]. This dilemma was due in part to some studies showing that, in contrast to adults, up to 79% of children infected with SARS-CoV-2 may be asymptomatic or paucisymptomatic (that is, subclinical), and could act as vectors for the spread and continuous circulation of the virus between schools and their families [124,125].

However, contrary to expectations, even in areas of Italy, England, the United States and Australia, where the number of community cases was high, the incidence of COVID-19 did not increase with the reopening of schools and day-care centers. Moreover, when outbreaks of the disease occurred in these locations, they affected few people; in particular, the school staff [126]. There is also some additional evidence that children may play a limited role in the transmission of SARS-CoV-2: a meta-analysis conducted by Viner et al. [127], including studies comprising children, adolescents and adults with laboratory diagnosis of COVID-19, showed that children and adolescents are less susceptible to SARS-CoV-2, with an odds ratio of 0.56 of being an infected contact compared to adults. On the other hand, a recent study in the USA found a large effect in reducing transmission for closing schools and universities in conjunction, but the study cannot distinguish their individual effects. Primary schools may be generally less affected than secondary schools, partly because children under 12 years old are less susceptible to SARS-CoV-2 infection, as previously discussed [128].

As discussed earlier, age did not impact viral load, but younger children have a lower ACE2 expression and a more robust innate immune response to SARS-CoV-2 [9]. In any case, asymptomatic children can be contagious, and the identification and management of this group of patients must be an integral part of the approach against the COVID-19 epidemic. Therefore, asymptomatic patients should be investigated through active search during COVID-19 outbreaks or screening for potential sources of infection, such as in schools [129,130].

## 7. Vaccines

COVID-19 is a potentially preventable disease. However, as with other novel pathogens, humanity is virtually disarmed against SARS-CoV-2 due to having no previous specific immune response to the pathogen. Vaccines have traditionally been considered a form of preventive intervention for direct and indirect protection (i.e., herd immunity) in a target population, but it is relevant to note that immunization is modulated by vaccine type, the individual response, adherence to prevention programs and age of administration [131]. According to the WHO landscape of COVID-19 vaccines [132], more than 236 candidates are under development, with 63 in clinical evaluation and 173 in the preclinical phase.

Vaccine technology platforms for COVID-19 include: live attenuated virus, inactivated virus, recombinant protein subunit, peptide-based, virus-like particle, non-replicating viral vector, replicating viral vector, DNA and mRNA. Each platform has advantages and disadvantages, related to its ability to induce potent immune responses, manufacturing capacity, and safety for clinical use, and it is still unclear which vaccine strategies will be most successful. New technologies, such as viral vector, DNA and mRNA are of simple design and can be produced at pandemic speed but, until recently, have never resulted in a licensed vaccine [2,133].

To meet the urgent need for a vaccine in a pandemic setting, a new strategy was proposed with multiple development steps in parallel, compressing the development timeline to 1–2 years from the usual 10–15 years. Nevertheless, any indication of a lack of safety in this process could fuel the anti-vaccination movement, jeopardizing the herd immunity aim. Due to this concern, the vast majority of clinical trials so far have not included children, seniors and those with underlying medical conditions, precisely the groups considered a priority for vaccination and with special safety issues [133,134].

Although the priority for COVID-19 vaccination would logically target those at the highest risk of infection (health care workers) and severe disease (older adults and patients with co-morbidities), vaccination of children may be considered another critical step, for their own protection and to support herd immunity. This is particularly true in countries with younger populations, such as in Africa [76]. Achieving herd immunization by an effective and safe vaccine will be a welcomed pathway to protect the population, stopping this pandemic, and vaccinating children may prove to be fundamental for this goal.

## 8. Conclusions

Despite the fast advances at this moment, there are more questions than answers about SARS-CoV-2 infection, including the remaining open question as to why COVID-19 is milder in children. Among the main hypotheses discussed in this review, more robust innate immunity and the lower expression of SARS-CoV-2 receptors in relation to adults seem the most attractive. On the other hand, SARS-CoV-2 has not been implicated as an important driver of viral wheeze or asthma exacerbations in allergic children. This fact seems to apply even to the childhood asthma–obesity phenotype. Despite this, children with severe and uncontrolled asthma should be considered at higher risk for the development of severe COVID-19. Focus on risk stratification and controller medication adherence will be essential to allow children with asthma to return safely to school.

In response to the COVID-19 pandemic, the international scientific community has developed a huge body of research knowledge in record time through a level of mobilization and cooperation that is unprecedented for an infectious disease. As a result of this translational approach, guidelines for mitigating the spread of the infection, practical diagnostic investigation tools, therapeutic approaches and vaccines have been developed. Hopefully, these and other pressing questions will be answered soon.

## Figures and Tables

**Table 1:** Clinical characteristics of SARS-CoV-2 infection in children and adults.

	Children	Adults
Asymptomatic or mild illness	More likely	Less likely
Main presentation symptoms (e.g., fever, cough, shortness of breath)	Similar	Similar
Upper respiratory tract involvement	Predominantly	Present
Lower respiratory tract involvement	Less common	Frequent
Wheezing	Infrequent	Infrequent
ARDS	Infrequent (mainly < 1 year old)	Possible (mainly > 65 years old)
Rate of co-infection with other respiratory viruses	Higher	Lower
Chest radiographic changes	Generally less pronounced	Pronounced in most cases
Neurological disorders and coagulopathy	Unknown prevalence	Present
Increased inflammation markers in severe cases	Yes	Yes
Severe Systemic Inflammatory Response Syndrome	Yes, as MIS-C	Yes, as Cytokine Storm
Mortality	Lower	Higher

MIS-C: Multisystem Inflammatory Syndrome; ARDS: Acute Respiratory Distress Syndrome.

## References

[1] Ludvigsson J.F. (2020). Systematic review of COVID-19 in children shows milder cases and a better prognosis than adults. Acta Paediatr..

[2] Boechat J.L., Chora I., Delgado L. (2020). Immunology of Coronavirus-19 Disease (COVID-19): A perspective for the clinician in the first 4 months of the emergence of SARS-CoV-2. Med. Interna.

[3] Cai J., Xu J., Lin D., Yang Z., Xu L., Qu Z., Zhang Y., Zhang H., Ran J., Liu P. (2020). A case series of children with 2019 novel coronavirus infection: Clinical an epidemiological features. Clin. Infect. Dis..

[4] WHO Director-General World Health Organization Opening Remarks at the Media Briefing on COVID-19. https://www.who.int/director-general/speeches/detail/who-director-general-s-opening-remarks-at-the-media-briefing-on-covid-19---11-march-2020.

[5] WHO Coronavirus Disease (COVID-19) Dashboard. https://covid19.who.int/.

[6] Castro-Rodriguez J.A., Forno E. (2020). Asthma and COVID-19 in children: A systematic review and call for data. Pediatric. Pulmonol..

[7] Tsankov B.K., Allaire J.M., Irvine M.A., Lopez A.A., Sauvé L.J., Vallance B.A., Jacobson K. (2021). Severe COVID-19 infection and pediatric comorbidities: A systematic review and Meta-Analysis. Int. J. Infect. Dis..

[8] Willians N., Radia T., Harman K., Agrawal P., Cook J., Gupta A. (2020). COVID-19 severe acute respiratory syndrome coronavirus 2 (SARS-CoV-2) infection in children and adolescents: A systematic review of critically unwell children and the association with underlying comorbidities. Eur J. Ped..

[9] Yonker L.M., Neilan A.M., Bartsch Y., Patel A.B., Regan J., Arya P., Gootkind E., Park G., Hardcastle M., St John A. (2020). Pediatric severe acute respiratory syndrome coronavirus 2 (SARS-CoV-2): Clinical presentation, infectivity, and immune responses. J. Pediatr..

[10] Dong Y., Mo X., Hu Y., Qi X., Jiang F., Jiang Z., Tong S. (2020). Epidemiology of COVID-19 among children in China. Pediatrics.

[11] Han M.S., Choi E.H., Chang S.H., Jin B.-L., Lee E.J., Kim B.N., Kim M.K., Doo K., Seo J.-H., Kim Y.-J. (2020). Clinical characteristics and viral RNA detection in children with Coronavirus Disease 2019 in the Republic of Korea. JAMA Pediatr..

[12] Yoon S., LI H., Lee K.H., Hong S.H., Kim D., Im H., Rah W., Kim E., Cha S., Yang J. (2020). Clinical characteristics of asymptomatic and symptomatic pediatric Coronavirus Disease 2019 (COVID-19): A systematic review. Medicina.

[13] Jutzeler C.R., Bourguignon L., Weis C.V., Tong B., Wong C., Rieck B., Pargger H., Tschudin-Sutter S., Egli A., Borgwardt K. (2020). Comorbidities, clinical signs and symptoms, laboratory findings, imaging features treatment strategies, and outcomes in adult and pediatric patients with COVID-19: A systematic review and meta-analysis. Travel Med. Infect. Dis..

[14] Rajapakse N., Dixit D. (2020). Human and novel coronavirus infections in children: A review. Paediatr. Int. Child. Health.

[15] Patel N.A. (2020). Pediatric COVID-19: Systematic review of the literature. Am. J. Otolaryngol..

[16] Cruz A.T., Zeichner S.L. (2020). COVID-19 in children: Initial characterization of the pediatric disease. Pediatrics.

[17] Rabha A.C., Oliveira F.I., Oliveira T.A., Cesar R.G., Fongaro G., Mariano R.F., Camargo C.N., Fernandes F.R., Wandalsen G.F. (2021). Clinical manifestations of children and adolescents with COVID-19: Report of the first 115 cases from Sabará Hospital Infantil. Rev. Paul. Pediatr..

[18] Kaye R., Chang C.W.D., Kazahaya K., Brereton J., Denneny J.C. (2020). COVID-19 anosmia reporting tool: Initial findings. Otolaryngol. Head Neck Surg..

[19] Mak P.Q., Chung K.S., Wong J.S., Shek C.C., Kwan M.Y. (2020). Anosmia and ageusia: Not an uncommon presentation of COVID-19 infection in children and adolescents. Pediatr Infect. Dis. J..

[20] Zhou M.-Y., Xie X.-L., Peng Y.-G., Wu M.-J., Deng X.-Z., Wu Y., Xiong L.-J., Shang L.-H. (2020). From SARS to COVID-19: What we have learned about children infected with COVID-19. Int. J. Inf. Dis..

[21] anonymous (2020). Long COVID: Let patients help define long-lasting COVID symptoms. Nature.

[22] Alwan N.A., Burgess R.A., Ashworth S., Beale R., Bhadelia N., Bogaert D., Dowd J., Eckerle I., Goldman L.R., Greenhalgh T. (2020). Scientific consensus on the COVID-19 pandemic: We need to act now. Lancet.

[23] Feldstein L.R., Rose E.B., Horwitz S.M., Collins J.P., Newhams M.M., Son M.B.F., Newburger J.W., Kleinman L.C., Heidemann S.M., Martin A.A. (2020). Multisystem inflammatory syndrome in U.S. children and adolescents. N. Engl. J. Med..

[24] Rivas M.N., Porritt R.A., Cheng M.H., Bahar I., Arditi M. (2020). COVID-19-associated multisystem inflammatory syndrome in children (MIS-C): A novel disease that mimics toxic shock syndrome—The superantigen hypothesis. J. Allergy Clin. Immunol..

[25] CDC Health Alert Network Multisystem Inflammatory Syndrome in Children (MIS-C) Associated with Coronavirus Disease 2019 (COVID-19). https://emergency.cdc.gov/han/2020/han00432.asp.

[26] Lee P.Y., Day-Lewis M., Henderson L.A., Friedman K.G., Lo J., Roberts J.E., Lo M.S., Platt C.D., Chou J., Hoyt K.J. (2020). Distinct clinical and immunological features of SARS-CoV-2-induced multisystem inflammatory syndrome in children. J. Clin. Investig..

[27] Verdoni L., Mazza A., Gervasoni A., Martelli L., Ruggeri M., Ciuffreda M., Bonanomi E., D’Antiga L. (2020). An outbreak of severe Kawasaki-like disease at the Italian epicentre of the SARS-COV-2 epidemic: An observational cohort study. Lancet.

[28] Viner R.M., Whittaker E. (2020). Kawasaki-like disease: Emerging complication during the Covid-19 pandemic. Lancet.

[29] Waltuch T., Gill P., Zinns L.E., Whitney R., Tokarski J., Tsung J.W., Sanders J.E. (2020). Features of COVID-19 post-infectious cytokine release syndrome in children presenting to the emergence department. Am. J. Emerg. Med..

[30] Toubiana J., Poirault C., Corsia A., Bajolle F., Fourgeaud J., Angoulvant F., Debray A., Basmaci R., Salvador E., Biscardi S. (2020). Kawasaki-like multisystem inflammatory syndrome in children during the Covid-19 pandemic in Paris, France: Prospective observational study. BMJ.

[31] Nakra N.A., Blumberg D.A., Herrera-Guerra A., Lakshminrusimha S. (2020). Multi-system inflammatory syndrome in children (MIS-C) following SARS-CoV-2 infection: Review of clinical presentation, hypothetical pathogenesis and proposed management. Children.

[32] Rowley A.H., Shulman S.T., Arditi M. (2020). Immune pathogenesis of COVID-19-related multisystem inflammatory syndrome in children. J. Clin. Investig..

[33] Suratannon N., Dik W.A., Chatchatee P., van Hagen P.M. (2020). COVID-19 in children: Heterogeneity within the disease and hypothetical pathogenesis. Asian Pac. J. Allergy Immunol..

[34] Götzinger F., Santiago-García B., Noguera-Julián A., Lanaspa M., Lancella L., Carducci F., Gabrovska N., Velizarova S., Prunk P., Osterman V. (2020). COVID-19 in children and adolescents in Europe: A multinational, multicentre cohort study. Lancet Child Adolesc. Health.

[35] Shelmerdine S.C., Lovrenski J., Caro-Domínguez P., Toso S., Collaborators of the European Society of Paediatric Radiology Cardiothoracic Imaging Taskforce (2020). Coronavirus disease 2019 (COVID-19) in children: A systematic review of imaging findings. Pediatr. Radiol..

[36] Xia W., Shao J., Guo Y., Peng X., Li Z., Hu D. (2020). Clinical and CT features in pediatric patients with COVID-19 infection: Different points from adults. Pediatr. Pulmonol..

[37] Cui X., Zhao Z., Zhang T., Guo W., Guo W., Zheng J., Zhang J., Dong C., Na R., Zheng L. (2021). A systematic review and meta-analysis of children with coronavirus disease 2019 (COVID-19). J. Med. Virol..

[38] Ghayda R.A., Lee J., Lee J.Y., Kim D.K., Lee K.H., Hong S.H., Han Y.J., Kim J.S., Yang J.W., Kronbichler A. (2020). Correlations of clinical and laboratory characteristics of COVID-19: A systematic review and meta-analysis. Int. J. Environ. Res. Public Health.

[39] Zhang L., Peres T.G., Silva M.V.F., Camargos P. (2020). What we know so far about Coronavirus Disease 2019 in children: A meta-analysis of 551 laboratory-confirmed cases. Pediatr. Pulmonol..

[40] Chao J.Y., Derespina K.R., Herold B.C., Goldman D.L., Aldrich M., Weingarten J., Ushay H.M., Cabana M.D., Medar S.S. (2020). Clinical characteristics and outcomes of hospitalized and critically ill children and adolescents with Coronavirus Disease 2019 at a tertiary care medical center in New York City. J. Pediatr..

[41] Ahmed M., Advani S., Moreira A., Zoretic S., Martinez J., Chorath K., Acosta S., Naqvi R., Burmeister-Morton F., Burmeister F. (2020). Multisystem inflammatory syndrome in children: A systematic review. EClinicalMedicine.

[42] Snape M.D., Viner R.M. (2020). COVID-19 in children and young people. Science.

[43] Patel A.B., Verma A. (2020). Nasal ACE2 levels and COVID-19 in children. JAMA.

[44] Bunyavanich S., Do A., Vicencio A. (2020). Nasal gene expression of angiotensin-converting enzyme 2 in children and adults. JAMA.

[45] Hoffmann M., Kleine-Weber H., Schroeder S., Krüger N., Herrler T., Erichsen S., Schiergens T.S., Herrler G., Wu N.-H., Nitsche A. (2020). SARS-CoV-2 cell entry depends on ACE2 and TMPRSS2 and is blocked by a clinically proven protease inhibitor. Cell.

[46] Yan R., Zhang Y., Li Y., Xia L., Guo Y., Zhou Q. (2020). Structural basis for the recognition of SARS-CoV-2 by full-length human ACE2. Science.

[47] Wang K., Chen W., Zhang Z.., Deng Y., Lian J.-Q., Du P., Wei D., Zhang Y., Sun X.-X., Gong L. (2020). CD147-spike protein is a novel route for SARS-CoV-2 infection to host cells. Signal Transduct Target Ther..

[48] Radzikowska U., Ding M., Tan G., Zhakparov D., Peng Y., Wawrzyniak P., Wang M., Li S., Morita H., Altunbulakli C. (2020). Distribution of ACE2, CD147, CD26 and other SARS-CoV-2 associated molecules in tissues and immune cells in health and in asthma, COPD, obesity, hypertension, and COVID-19 risk factors. Allergy.

[49] Wu Q., Xing Y., Shi L., Li W., Gao Y., Pan S., Wang Y., Wang W., Xing Q. (2020). Coinfection and other clinical characteristics of COVID-19 in children. Pediatrics.

[50] Nickbakhsh S., Mair C., Matthews L., Reeve R., Johnson P.C.D., Thorburn F., von Wissmann B., Reynolds A., McMenamin J., Gunson R.N. (2019). Virus-virus interactions impact the population dynamics of influenza and the common cold. Proc. Natl. Acad. Sci. USA.

[51] Casalegno J.S., Ottmann M., Duchamp M.B., Escuret V., Billaud G., Frobert E., Morfin F., Lina B. (2010). Rhinoviruses delayed the circulation of the pandemic influenza A (H1N1) 2009 virus in France. Clin. Microbiol. Infect..

[52] Mak G.C., Wong A.H., Ho W.Y., Lim W. (2012). The impact of pandemic influenza A (H1N1) 2009 on the circulation of respiratory viruses 2009-2011. Influenza Other Res. Viruses.

[53] Ma L., Wang W., Le Grange J.M., Wang X., Du S., Li C., Wei J., Zhang J.-N. (2020). Coinfection of SARS-CoV-2 and Other Respiratory Pathogens. Infect. Drug Resist..

[54] Du H., Dong X., Zhang J.-J., Cao Y.-Y., Akdis M., Huang P.-Q., Chen H.-W., Li Y., Liu G.-H., Akdis C.A. (2020). Clinical characteristics of 182 pediatric COVID-19 patients with different severities and allergic status. Allergy.

[55] Simon A.K., Hollander G.A., McMichael A. (2015). Evolution of the immune system in humans from infancy to old age. Proc. R. Soc. B.

[56] Andreakos E., Zanoni I., Galani I.E. (2019). Lambda interferons come to light: Dual function cytokines mediating antiviral immunity and damage control. Curr. Opin. Immunol..

[57] Tomaiuolo R., Ruocco A., Salapete C., Carru C., Baggio G., Franceschi C., Zinellu A., Vaupel J., Bellia C., Lo Sasso B. (2012). Activity of mannose-binding lectin in centenarians. Aging Cell.

[58] Matricardi P.M., Dal Negro R.W., Nisini R. (2020). The first, holistic immunological model of COVID-19: Implications for prevention, diagnosis, and public health measures. Pediatr. Allergy Immunol..

[59] Maggi E., Canonica G.W., Moretta L. (2020). COVID-19: Unanswered questions on immune response and pathogenesis. J. Allergy Clin. Immunol..

[60] Carsetti R., Quintarelli C., Quinti I., Mortari E.P., Zumla A., Ippolito G., Locatelli F. (2020). The immune system of children: The key to understanding SARS-CoV-2 susceptibility?. Lancet Child Adolesc. Health.

[61] Licari A., Votto M., Brambilla I., Castagnoli R., Piccotti E., Olcese R., Tosca M.A., Ciprandi G., Marseglia G.L. (2020). Allergy and asthma in children and adolescents during the COVID outbreak: What we know and how we could prevent allergy and asthma flares. Allergy.

[62] Netea M.G., Dominguez-Andres. J., Barreiro L.B., Chavakis T., Divangahi M., Fuchs E., Joosten L.A.B., van der Meer J.W.M., Mhlang M.M., Mulder W.J.M. (2020). Defining trained immunity and its role in health and disease. Nat. Rev. Immunol..

[63] Grifoni A., Weiskopf D., Ramirez S.I., Mateus J., Dan J.M., Moderbacher C.R., Rawlings S.A., Sutherland A., Premkumar L., Jadi R.S. (2020). Targets of T cell responses to SARS-CoV-2 coronavirus in humans with COVID-19 disease and unexposed individuals. Cell.

[64] Sette A., Crotty S. (2020). Pre-existing immunity to SARS-CoV-2: The knowns and unknowns. Nat. Rev. Immunol..

[65] Chen Z., Wherry E.J. (2020). T cell responses in patients with COVID-19. Nat. Rev. Immunol..

[66] Kellam P., Barclay W. (2020). The dynamics of humoral immune responses following SARS-CoV-2 infection and the potential for reinfection. J. Gen. Virol..

[67] Roy S. (2020). COVID-19 Reinfection: Myth or Truth?. SN Compr. Clin. Med..

[68] Osman A.A., Al Daajani M.M., Alsahafi A.J. (2020). Re-positive coronavirus disease 2019 PCR test: Could it be a reinfection?. New Microbes New Infect..

[69] Kimura H., Francisco D., Conway M., Martinez F., Vercelli D., Polverino F., Billheimer D., Kraft M. (2020). Type 2 inflammation modulates ACE2 and TMPRSS2 in airway epithelial cells. J. Allergy Clinimmunol..

[70] Ziegler C.G.K., Allon S.J., Nyquist S.K., Mbano I.M., Miao V.N., Tzouanas C.N., Cao Y., Yousif A.F., Bals J., Hauser B.M. (2020). SARS-CoV-2 receptor ACE2 is an interferon-stimulated gene in human airway epithelial cells and is detected in specific cell subsets across tissues. Cell.

[71] Sokolowska M., Lukasik Z.M., Agache I., Akdis C.A., Akdis D., Akdis M., Barcik W., Brough H.A., Eiwegger T., Eljaszewicz A. (2020). Immunology of COVID-19: Mechanisms, clinical outcome, diagnostics and perspectives—A report of the European Academy of Allergy and Clinical Immunology (EAACI). Allergy.

[72] Topley W.W., Wilson G.S. (1923). The spread of bacterial infection: The problem of herd-immunity. J. Hyg..

[73] Xia Y., Zhong L., Tan J., Zhang Z., Lyu J., Chen Y., Zhao A., Huang L., Long Z., Liu N.-N. (2020). How to understand “herd immunity” in Covid-19 pandemic. Front. Cell Dev. Biol..

[74] Nikolai L.A., Meyer C.G., Kremsner P.G., Velavan T.P. (2020). Asymptomatic SARS Coronavirus 2 infection: Invisible yet invincible. Int. J. Infect. Dis..

[75] Kwok K.O., Lai F., Wei W.I., Wong S.Y., Tang J.W. (2020). Herd immunity—Estimating the level required to halt the COVID-19 epidemics in affected countries. J. Infect..

[76] Velavan T.P., Pollard A.J., Kremsner P.G. (2020). Herd immunity and vaccination of children for COVID-19. Int. J. Infect. Dis..

[77] Johnston S.L. (2005). Overview of virus-induced airway disease. Proc. Am. Thorac. Soc..

[78] Heimdal I., Moe N., Krokstad S., Christensen A., Skanke L.H., Nordbo S.A., Dollner H. (2019). Human coronavirus in hospitalized children with respiratory tract infections: A 9-year population-based study from Norway. J. Infect. Dis..

[79] Zeng Z.-Q., Chen D.-H., Tan W.-P., Qiu S.-Y., Xu D., Liang H.-X., Chen M.-X., Li X., Lin Z.-S., Liu W.-K. (2018). Epidemiology and clinical characteristics of human coronaviruses OC43, 229E, NL63, and HKU1: A study of hospitalized children with acute respiratory tract infection in Guangzhou, China. Eur. J. Clin. Microbiol. Infect. Dis..

[80] Varghese L., Zachariah P., Vargas C., LaRussa P., Demmer R.T., Furuya Y.E., Whittier S., Reed C., Stockwell M.S., Saiman L. (2018). Epidemiology and clinical features of human coronaviruses in the pediatric population. J. Pediatr. Infect. Dis. Soc..

[81] Shirato K., Imada Y., Kawase M., Nakagaki K., Matsuyama S., Taguchi F. (2014). Possible involvement of infection with human coronavirus 229E, but not NL63, in Kawasaki disease. J. Med. Virol..

[82] Chang L.-Y., Chiang B.-L., Kao C.-L., Wu M.-H., Chen P.-J., Berkhout B., Yang H.-C., Huang L.-M., Kawasaki Disease Research Group (2006). Lack of association between infection with a novel human coronavirus (HCoV), HCoV-NH, and Kawasaki disease in Taiwan. J. Infect. Dis..

[83] Stockman L.J., Massoudi M.S., Helfand R., Erdman D., Siwek A.M., Anderson L.J., Parashar U.D. (2007). Severe acute respiratory syndrome in children. Pediatr. Infect. Dis. J..

[84] Leung C.-W., Kwan Y.-W., Ko P.-W., Chiu S.S., Loung P.-Y., Fong N.-C., Lee L.-P., Hui Y.-W., Law H.K.W., Wong W.H.S. (2004). Severe acute respiratory syndrome among children. Pediatrics.

[85] Thabet F., Chehab M., Bafaqih H., Mohaimeed S.A. (2015). Middle East respiratory syndrome coronavirus in children. Saudi. Med. J..

[86] Wu A., Peng Y., Huang B., Ding X., Wang X., Niu P., Meng J., Zhu Z., Zhang Z., Wang J. (2020). Genome composition and divergence of the novel coronavirus (2019-nCoV) originating in China. Cell Host Microbe.

[87] Skevaki C., Karsonova A., Karaulov A., Xie M., Renz H. (2020). Asthma-associated risk for COVID-19 development. J. Allergy Clin. Immunol..

[88] Solé D., Wandalsen G., Camelo-Nunes I., Naspitz C., ISAAC—Brazilian Group (2006). Prevalence of symptoms of asthma, rhinitis, and atopic eczema among Brazilian children and adolescents identified by the International Study of Asthma and Allergies in Childhood (ISAAC)—Phase 3. J. Pediatr. (Rio J.).

[89] Papadopoulos N., Custovic A., Deschildre A., Mathioudakis A., Phipatanakul W., Wong G., Xepapadaki P., Agache I., Bacharier L., Bonini M. (2020). Impact of COVID-19 on pediatric asthma: Practice adjustments and disease burden. J. Allergy Clin. Immunol. Pract..

[90] Ruano F., Álvarez M., Haroun-Díaz E., de la Torre M., González P., Prieto-Moreno A., Rojas I.T., García M.D.C., Alzate D.P., Blanca-Lopez N. (2020). Impact of the COVID-19 pandemic in children with allergic asthma. J. Allergy Clin. Immunol. Pract..

[91] Carli G., Cecchi L., Stebbing J., Parronchi P., Farsi A. (2020). Is asthma protective against COVID-19?. Allergy.

[92] Roland D., Teo K.W., Bandi S., Lo D., Gaillard E. (2020). COVID-19 is not a driver of clinically significant viral wheeze and asthma. Arch. Dis. Child..

[93] Chavasse R., Almario A., Christopher A., Kappos A., Shankar A. (2020). The indirect impact of COVID-19 on children with asthma. Arch. Bronconeumol..

[94] Abrams E.M., Szefler S.J. (2020). Managing asthma during Coronavirus Disease-2019: An example for other chronic conditions in children and adolescents. J. Pediatr..

[95] Brough H.A., Kalayci O., Sediva A., Untersmayr E., Munblit D., Rodriguez Del Rio P., Vazquez-Ortiz M., Arasi S., Alvaro-Lozano M., Tsabouri S. (2020). Managing childhood allergies and immunodeficiencies during respiratory virus epidemics—The 2020 COVID-19 pandemic: A statement from the EAACI-section on pediatrics. Pediatr. Allergy Immunol..

[96] Jackson D., Busse W., Bacharier L., Kattan M., O’Connor G., Wood R., Visness C.M., Durham S.R., Larson D., Esnault S. (2020). Association of respiratory allergy, asthma, and expression of the SARS-CoV-2 receptor ACE2. J. Allergy Clin. Immunol..

[97] Yang J., Koh H., Moon S., Yoo I., Ha E., You S., Kim S.Y., Yon D.K., Lee S.W. (2020). Allergic disorders and susceptibility to and severity of COVID-19: A nationwide cohort study. J. Allergy Clin. Immunol..

[98] Du Y., Tu L., Zhu P., Mu M., Wang R., Yang P., Wang X., Hu C., Ping R., Hu P. (2020). Clinical features of 85 fatal cases of COVID-19 from Wuhan. A retrospective observational study. Am. J. Respir. Crit. Care Med..

[99] Li Q., Ding X., Xia G., Chen H.-G., Chen F., Geng Z., Xu L., Lei S., Pan A., Wang L. (2020). Eosinopenia and elevated C-reactive protein facilitate triage of COVID-19 patients in fever clinic: A retrospective case-control study. EClinicalMedicine.

[100] Azkur A.K., Akdis M., Azkur D., Sokolowska M., van de Veen W., Brüggen M.-C., O’Mahoni L., Gao Y., Nadeau K., Akdis C.A. (2020). Immune response to SARSCoV-2 and mechanisms of immunopathological changes in COVID-19. Allergy.

[101] Yates T., Razieh C., Zaccardi F., Davies M.J., Khunti K. (2020). Obesity and risk of COVID-19: Analysis of UK biobank. Prim. Care Diabetes.

[102] Lovinsky-Desir S., Deshpande D.R., De A., Murray L., Stingone J.A., Chan A., Patel N., Rai N., DiMango E., Milner J. (2020). Asthma among hospitalized patients with COVID-19 and related outcomes. J. Allergy Clin. Immunol..

[103] Chiappetta S., Sharma A.M., Bottino V., Stier C. (2020). COVID-19 and the role of chronic inflammation in patients with obesity. Int J. Obes. (Lond).

[104] World Health Organization, United Nations Children’s Fund, World Bank Levels and Trends in Child Malnutrition: UNICEFWHO-World Bank Joint Child Malnutrition Estimates. https://www.who.int/nutgrowthdb/estimates/en/.

[105] Dixon A.E., Holguin F. (2019). Diet and metabolism in the evolution of asthma and obesity. Clin. Chest Med..

[106] Barros R., Delgado L. (2016). Visceral adipose tissue: A clue to the obesity-asthma endotype(s)?. Rev. Port. Pneumol..

[107] Hosoki K., Chakraborty A., Sur S. (2020). Molecular mechanisms and epidemiology of COVID-19 from an allergist’s perspective. J. Allergy Clin. Immunol..

[108] Bousquet J., Jutel M., Akdis C.A., Klimek L., Pfaar O., Nadeau K.C., Eiwegger T., Bedbrook A., Ansotegui I.J., Anto J.M. (2020). ARIA-EAACI statement on asthma and COVID-19 (June 2, 2020). Allergy.

[109] Grace J., Mohan A., Lugogo N.L. (2019). Obesity and adult asthma: Diagnostic and management challenges. Curr. Opin. Pulm. Med..

[110] Madeira L.N.O., Bordallo M.A.N., Borges M.A., Lopes A.J., Madeira I.R., Kuschnir F.C. (2020). Relations between asthma and obesity: An analysis of multiple factors. Rev. Paul. Pediatr..

[111] Matsuyama S., Kawase M., Nao N., Shirato K., Ujike M., Kamitani W., Shimojima M., Fukushi S. (2020). The inhaled steroid ciclesonide blocks SARS-CoV-2 RNA replication by targeting the viral replication-transcription complex in cultured cells. J. Virol..

[112] Barré J., Sabatier J., Annweiler C. (2020). Montelukast drug may improve COVID-19 prognosis: A review of evidence. Front. Pharmacol..

[113] Yamaya M., Nishimura H., Deng X., Sugawara M., Watanabe O., Nomura K., Shimotai Y., Momma H., Ichinose M., Kawase T. (2020). Inhibitory effects of glycopyrronium, formoterol, and budesonide on coronavirus HCoV-229E replication and cytokine production by primary cultures of human nasal and tracheal epithelial cells. Respir. Investig..

[114] Peters M., Sajuthi S., Deford P., Christenson S., Rios C., Montgomery M., Woodruff P.G., Mauger D.T., Erzurum S.C., Johansson M.W. (2020). COVID-19–related genes in sputum cells in asthma: Relationship to demographic features and corticosteroids. Am. J. Respir. Crit. Care Med..

[115] Halpin D., Singh D., Hadfield R. (2020). Inhaled corticosteroids and COVID-19: A systematic review and clinical perspective. Eur. Respir. J..

[116] Global Initiative for Asthma COVID-19: GINA Answers to Frequently Asked Questions on Asthma Management. www.ginasthama.org.

[117] Shaker M., Oppenheimer J., Grayson M., Stukus D., Hartog N., Hsieh E., Rider N., Dutmer C.M., Leek T.K.V., Kim H. (2020). COVID-19: Pandemic contingency planning for the allergy and immunology clinic. J. Allergy Clin. Immunol. Pract..

[118] WHO—World Health Organization (2020). Clinical Management of Severe Acute Respiratory Infection When COVID-19 Is Suspected.

[119] Hasan S., Capstick T., Zaidi S., Know C., Merchant H. (2020). Use of corticosteroids in asthma and COPD patients with or without COVID-19. Respir. Med..

[120] Tomazini B., Maia I., Cavalcanti A., Berwanger O., Rosa R., Veiga V., Avezum A., Lopes R.D., Bueno F.R., Silva M.V. (2020). Effect of dexamethasone on days alive and ventilator-free in patients with moderate or severe acute respiratory distress syndrome and COVID-19: The CoDEX randomized clinical trial. JAMA.

[121] Vultaggio A., Agache I., Akdis C., Akdis M., Bavbek S., Bossios A., Bousquet J., Boyman O., Chaker A.M., Chan S. (2020). Considerations on biologicals for patients with allergic disease in times of the COVID-19 pandemic: An EAACI statement. Allergy.

[122] Abrams E.M., Sinha I., Fernandes R.M., Hawcutt D.B. (2020). Pediatric asthma and COVID-19: The known, the unknown, and the controversial. Pediatr. Pulmonol..

[123] Centers for Disease Control and Prevention. https://www.cdc.gov/coronavirus/2019-ncov/need-extra-precautions/asthma.html.

[124] Davies N.G., Klepac P., Liu Y., Prem K., Jit M., Eggo R.M., CMMID COVID-19 Working Group (2020). Age-dependent effects in the transmission and control of COVID-19 epidemics. Nat. Med..

[125] Johansson M.A., Quandelacy T.M., Kada S., Prasad P.V., Steele M., Brooks J.T., Slayton R.B., Biggerstaff M., Butler J.C. (2021). SARS-CoV-2 transmission from people without COVID-19 symptoms. JAMA Netw. Open.

[126] Lewis D. (2020). Why schools probably aren’t COVID hotspots. Nature.

[127] Viner R.M., Mytton O.T., Bonell C., Melendez-Torres G.J., Ward J., Hudson L., Waddington C., Thomas J., Russell S., van der Klis F. (2020). Susceptibility to SARS-CoV-2 infection among children and adolescents compared with adults: A systematic review and meta-analysis. JAMA Pediatr..

[128] Brauner J.M., Mindermann S., Sharma M., Johnston D., Salvatier J., Gavenciak T., Stephenson A.B., Leech G., Altman G., Mikulik V. (2020). Inferring the effectiveness of government interventions against COVID-19. Science.

[129] Peng J., Su D., Zhang Z., Wang M. (2020). Identification and management of asymptomatic carriers of coronavirus disease 2019 (COVID-19) in China. Influenza Other Respir. Viruses.

[130] Rahimi F., TalebiBezminAbadi A. (2020). Challenges of managing the asymptomatic carriers of SARS-CoV-2. Travel Med. Infect. Dis..

[131] Clemente-Suárez V.J., Hormeño-Holgado A., Jiménez M., Benitez-Agudelo J.C., Navarro-Jiménez E., Perez-Palencia N., Maestre-Serrano R., Laborde-Cardenas C.C., Tornero-Aguilera J.F. (2020). Dynamics of population immunity due to the herd effect in the COVID-19 pandemic. Vaccines.

[132] WHO—World Health Organization Landscape of COVID-19 Candidates Vaccines. https://www.who.int/who-documents-detail/draft-landscape-of-covid-19-candidate-vaccines.

[133] Krammer F. (2020). SARS-CoV-2 vaccines in development. Nature.

[134] Jeyanathan M., Afkhami S., Smaill F., Miller M.S., Lichty B.D., Xing Z. (2020). Immunological considerations for COVID-19 vaccine strategies. Nat. Rev. Immunol..

